# I don’t understand how I feel: mediating role of impaired self-mentalizing in the relationship between childhood adversity and psychosis spectrum experiences

**DOI:** 10.3389/fpsyt.2023.1268247

**Published:** 2023-11-30

**Authors:** Jacqueline Nonweiler, Pilar Torrecilla, Thomas R. Kwapil, Sergi Ballespí, Neus Barrantes-Vidal

**Affiliations:** ^1^Departament de Psicologia Clínica i de la Salut, Universitat Autònoma de Barcelona, Barcelona, Spain; ^2^Department of Psychology, University of Illinois at Urbana-Champaign, Champaign, IL, United States; ^3^CIBER de Salud Mental, Instituto de Salud Carlos III, Madrid, Spain

**Keywords:** mentalization, self-other, schizotypy, parental loss, childhood adversity, paranoia, psychotic-like experiences

## Abstract

**Introduction:**

Childhood adversity is associated with the severity of multiple dimensions of psychosis, but the mechanisms underpinning the close link between the two constructs is unclear. Mentalization may underlie this relationship, as impaired mentalizing is found in various stages of the psychosis continuum. Nonetheless, the differential roles of self- and other-mentalizing in psychosis are not well understood.

**Methods:**

Parallel multiple mediation was conducted for the relationship between a diverse range of childhood adversity types, including intentional and nonintentional harm, and schizotypy (positive, negative, disorganized), psychotic-like experiences (PLE) and paranoia via self-mentalizing (attention to emotions and emotional clarity) and other-mentalizing in *n* = 1,156 nonclinically ascertained young adults.

**Results:**

Significant parallel multiple mediation models were found for all psychotic outcomes except negative schizotypy. The associations between intentionally harmful childhood adversity and psychotic outcomes were significantly mediated by increased attention to emotions for most models and decreased emotional clarity for some models. No significant mediation was found for parental loss. Paternal abuse was only mediated by attention to emotions whereas the effects of maternal abuse were mediated by attention to emotions and emotional clarity. Other-mentalizing only showed mediating effects on one of thirty models tested.

**Conclusion:**

Results highlight the mediating role of impaired self-mentalizing in the association between childhood adversity and psychosis. This is consistent with disturbances of self-concept and self-boundary characterizing, in particular, the positive dimension of psychosis. Maternal versus paternal figures may contribute differentially to the development of mentalizing. These results could inform future preventative interventions, focusing on the development and maintenance of self-mentalizing.

## 1 Introduction

Childhood adversity is associated with the severity of multiple domains of psychosis symptoms (1–4) and predicts later transition to psychosis (5). It encompasses a range of experiences including emotional, physical, and sexual abuse, along with emotional and physical neglect, and other ‘nonintentional’ adverse experiences that may occur during childhood such as the loss of a parent. Nonetheless, mechanisms underpinning the close link between the different types of childhood adversity and psychosis remain unclear. One mechanism that may underlie the relationship between childhood adversity and psychosis that is associated with both factors is mentalization, a multidimensional construct that incorporates the ability to notice and understand internal mental states of the self and others (6, 7). The role of mentalization in severe mental health problems such as personality disorders and psychosis has evolved to indicate that it is a transdiagnostic protective factor (8) that can be fostered across the developmental course to improve social, functional, and therapeutic outcomes and wellbeing.

The link between maltreatment and mentalizing is intuitive, as mentalizing is developed through social interaction in which understanding of complex social cues is mirrored from important attachment figures (e.g., parents) back to the child (9). Through identification of children’s mental states, parents help the child to develop understanding of their own mental states (10). In the case of childhood maltreatment, however, attachment relationships are often disrupted, and children may not be given, could dislike, or may even miss learning this crucial developmental skill entirely by avoiding reflection of the caregiver’s mental states (11, 12). Thus, childhood adversity can result in subsequent impairment or delayed development of the ability to mentalize (13–15), as well as discriminate and understand emotions (16–19).

Expanding the knowledge base of mentalization led to rationales for the role of this construct in the psychosis spectrum (20–23) and to proposed (24) and successful mentalization-based interventions for psychosis (25); however, the specific mechanistic relationships between mentalization and psychosis are not well understood (21, 26). Indeed, a novel area of inquiry is understanding the role of mentalization at sub-clinical levels. From a dimensional perspective, schizotypy is conceptualized as a broad phenotype that encompasses personality traits, subclinical expressions like psychotic-like experiences (PLE), and psychotic disorders (e.g., schizophrenia) (27). Consistent with the multidimensionality of psychosis, schizotypy is composed of at least three dimensions, namely, positive, negative and disorganized schizotypy, that have distinct associations with risk factors and associated symptoms similar to psychotic disorders (27–29). Subclinical schizotypy is consistently associated with PLE and psychosis symptoms, and the development of schizophrenia-spectrum disorders (30–32). Studying subclinical manifestations of psychosis helps to avoid the confounding effects associated with clinical status (e.g., symptom severity, medication effects, stigma, comorbidity, etc.), and thus enhances the analysis of etiological factors and mechanisms involved in the developmental course and trajectory of psychosis risk and resilience (33–35).

Research supports poor mentalization, usually operationalized using Theory of Mind tasks to evaluate understanding of others’ mental states, as a risk factor in several stages of the psychosis spectrum. Mentalizing impairments are found in earlier stages of the psychosis spectrum at attenuated levels (36). They are present in help-seeking groups who experience temporary psychotic states (37–41), and in community samples reporting PLE (42, 43). More severe expressions of the psychosis continuum, such as schizoaffective disorder (44) and, in particular, disorganized schizophrenia, are also associated with impaired mentalization (44, 45). Furthermore, mentalization has been shown to mediate the relationship between several risk factors and PLE (46), psychosis symptoms (47, 48), and psychotic disorders (49).

Studies examining the differential relationships of impaired mentalization with psychosis dimensions are scant and clear conclusions cannot be drawn. In clinical psychosis, the negative symptom dimension in general (50) and social dysfunction in particular (51, 52) have been associated with poor mentalization, but hypotheses that mentalization is related to the positive dimension are less often supported by evidence (48, 51). Nonetheless, this could be due to operationalization of mentalization typically focused on understanding others’ mental states, but not understanding of one’s own (i.e., self mentalizing), which may be more closely related to the self-identity and self-boundary disturbances that characterize the positive dimension (53, 54). Although associations between positive symptoms and mentalization have been found between sub-threshold hallucinations/delusions and poor performance on mentalizing tasks (42, 55, 56), contradicting evidence exists (48, 51, 57). Studies examining associations between subclinical disorganized schizotypy and mentalization have been limited and the results are equivocal; to our knowledge, only one study examines this relationship, which found that only social anxiety (negative dimension) and odd speech (disorganized dimension) were associated with impaired mentalization, which mediated the relationship between schizotypy and thought problems, an indicator of disorganized outcomes (58).

Whereas associations between aberrant mentalization and different levels of psychosis expression are established, to date, the great majority of mentalization research has considered the construct as a whole, despite mentalization being understood to operate under four primary dimensions; self-other; automatic-controlled, cognitive-affective, and internal-external (59). Recently, a call for increased focus on the dimensions of mentalizing and their distinct roles and significance in various spectral disorders has been posed (59). Research by our group evaluating self- and other-mentalization as mediators and moderators in mental health symptomatology indicates that self-mentalization is a particularly relevant factor (60–62). Although the role of the self has been a focus of psychosis spectrum research for decades (63–68), a paucity of psychosis research to date has focused on self-mentalization (69).

### 1.1 The present study

In an aim to integrate both the understudied disorganized schizotypy dimension and the dearth of self-mentalization evidence particularly in subclinical schizotypy, this study will explore the relationship of the self-other polarity of mentalization with the three schizotypy dimensions. First, we aim to explore the associations of self- and other-mentalization with positive, negative, and disorganized schizotypy in a nonclinically ascertained sample. Secondly, we will examine, for the first time, the possible mediating role of self-and other-mentalization in the relationship between a wide range of childhood adversity experiences with schizotypy, PLE, and paranoia. To our knowledge, only one study to date has examined the mediating role of mentalization in the relationship between childhood maltreatment and psychosis, albeit in a clinical sample, which revealed that mentalizing only mediated the relationship between childhood maltreatment and negative symptoms (48). Of note, different forms of intentional (e.g., maltreatment) and nonintentional (e.g., parental loss) adversity experiences, as well as the distinction between paternal versus maternal abuse during childhood, will be examined.

We predicted that positive, negative, and disorganized schizotypy would be associated with impaired self-mentalization. That is, individuals with high schizotypy would notice and understand their own emotions, thoughts, and feelings more poorly. While some evidence suggests that high positive and negative schizotypy dimensions are linked to deficits in emotional awareness and regulation (70), given the lack of clear grounding on the differential contributions of self-mentalizing factors versus other-mentalizing in schizotypy, and that most mentalization research in psychosis has only focused on other-mentalization, the study is exploratory regarding self-mentalization. For other-mentalization, we expected that the associations with schizotypy dimensions in this nonclinical sample would be aligned with previous research (i.e., negatively associated with other-mentalization) (36, 44, 45, 47, 71, 72), albeit at an attenuated level. Next, we expected that self- and other-mentalization would mediate the association between childhood maltreatment and all schizotypy dimensions, PLE and paranoia. Finally, following previous results found in epidemiological studies (73), prospective cohorts (74) and the group’s previous findings using experience sampling methodology (75), we hypothesized that the relationship between childhood adversity and psychotic outcomes via mentalizing would be more pronounced for those types of adversity characterized by an ‘intention to harm’ as compared to accidental adversity such as loss of a parent.

## 2 Methods

### 2.1 Participants

Participants were recruited at a university using posters and an email distributed to all students and university staff inviting them to take part in a broader study about environmental sensitivity and mental health (approved by the Ethics Committee of the Universitat Autònoma de Barcelona, ref. 5426). Participants were excluded if they were under 18 years old or had grandparents of non-Spanish origin, an exclusion criterion placed for the context of the broader study for genetic analysis purposes. After removing *n* = 47 participants of non-Spanish origin, *n* = 38 participants with careless responses according to the Infrequency Scale (76), and data from *n* = 7 dropout participants, responses from the original sample of *n* = 1,246 were reduced to *n* = 1,156 (*M_age_* = 23.29, SD = 6.49; range 18–62 years; 76.2% female). Of the final samle, *n* = 545 (47.1%) of participants had previously or were currently undergoing psychological treatment, and *n* = 204 (17.6%) had previously or were currently undergoing pharmacological treatment related to mental health.

### 2.2 Procedure

After obtaining informed consent, participants were administered an online questionnaire via Qualtrics survey software that included all materials of the present study. Participants were able to re-enter the questionnaire to complete it in multiple sessions if desired with a maximum allotted time for completion of 3 days.

### 2.3 Materials

#### 2.3.1 Childhood adversity

The Childhood Trauma Questionnaire-Brief (CTQ-B) (77) is a widely used self-reported measure with 28 items assessing the severity of sexual, physical and emotional abuse and physical and emotional neglect before the age of 18 years old. To reduce factors for childhood adversity, subscale totals for physical abuse, emotional abuse, physical neglect and emotional neglect were reduced to a single component for emotional/physical adversity. A detailed description of this procedure can be referenced in the ‘Data Analysis’ section.

The Childhood Care and Abuse Questionnaire-Brief (CECA.Q) (78–80) assesses aspects of childhood adversity that are not covered in the CTQ-B (e.g., parental loss, role reversal). It includes subscales for maternal antipathy, paternal antipathy, maternal psychological abuse, paternal psychological abuse, parental loss, role reversal and support. All CECA.Q subscales were included in the present study except the support subscale, which does not measure adversity.

#### 2.3.2 Mentalization

The recently developed Mentalization Scale (Ment-S) (81) was administered as it is the only mentalization questionnaire with a distinct factor for other-mentalization. The 10 items of the other-mentalization subscale were assessed and a total sum score was employed for the study. The Trait Meta-Mood Scale-24 (TMMS) (82) was administered to evaluate self-mentalization, as it offers two 8-item factors to further classify emotional self-knowledge: attention to emotions (8 items) and emotional clarity (8 items).

#### 2.3.3 Schizotypy, positive PLE and paranoid traits

The Multidimensional Schizotypy Scale-Brief (MSS-B) (83) is a 38-item self-report measure designed to assess the positive (MSS Positive; 13 items), negative (MSS Negative; 13 items) and disorganized (MSS Disorganized; 12 items) schizotypy dimensions. Evidence shows that this scale overcomes limitations of other schizotypy measures such as an unclear conceptual framework, outdated items, ethnic/sex differences, or exclusion of the disorganized dimension. The scale has good internal reliability and construct validity (28, 83).

PLE were measured using the frequency score of the positive subscale (20 items) of the Community Assessment of Psychic Experiences (CAPE) (84). Paranoia personality traits were assessed with the ideas of reference (9 items) and suspiciousness (8 items) subscales of the Schizotypal Personality Questionnaire (85).

### 2.4 Data analysis

Descriptive statistics, internal consistencies and correlational analysis were conducted for all variables of interest (Table 1). Note that some subscales differ slightly in their number of respondents. For CAPE Positive PLE and MSS Disorganized Schizotypy, missing data is due to a technical error in the data collection software. For CECA.Q psychological abuse and antipathy, reduced responses are reflective of the number of respondents who only had a paternal or a maternal figure, but not both. Sample size for CECA.Q antipathy is in all cases smaller compared to psychological abuse because one item of this subscale was only responded by participants who have siblings.

**Table 1:** Descriptive statistics.

	α	Mean/SD	Min	Max	Skewness	Kurtosis
TMMS attention	0.89	27.84/7.32	9	40	−0.28	−0.78
TMMS clarity	0.93	25.84/7.48	9	40	0.04	−0.83
Ment-S other mentalizing	0.79	40.55/5.03	20	50	−0.50	0.28
**Childhood adversity (CTQ)**						
Emotional abuse	0.83	8.88/4.30	5	25	1.40	1.65
Physical abuse	0.73	5.64/1.63	5	20	4.10	22.03
Emotional neglect	0.86	10.44/4.26	5	25	0.63	−0.29
Physical neglect	0.46	6.26/1.92	5	19	2.24	6.89
**Childhood adversity (CECA.Q)**						
Parental loss	0.27^a^	0.22/0.49	0	3	0.07	4.56
Psychological abuse (father) (*n* = 996)^b^	0.82	2.08/3.20	0	16	1.93	3.45
Antipathy (father) (*n* = 996)^b^	0.74	2.31/2.81	0	12	1.39	1.14
Psychological abuse (mother) (*n* = 1,105)^b^	0.78	3.14/3.64	0	17	1.27	1.00
Antipathy (mother) (*n* = 1,105)^b^	0.81	1.72/2.77	0	12	1.78	2.40
Paternal abuse (*n* = 996)^b^	–^c^	0.00/0.94	−0.74	3.74	1.69	2.32
Maternal abuse (*n* = 1,105)^b^	–^c^	0.00/0.94	−0.86	3.58	1.52	1.70
Role reversal	0.79	11.64/7.09	0	32	0.53	−0.34
CAPE positive PLE (*n* = 1,111)^b^	0.83	30.31/6.37	20	58	0.93	0.82
MSS positive schizotypy	0.76	2.76/2.68	0	12	0.95	0.21
MSS negative schizotypy	0.70	2.12/2.15	0	12	1.56	2.89
MSS disorganized schizotypy (*n* = 1,115)^b^	0.88	3.56/3.56	0	12	0.79	−0.59
SPQ suspiciousness	0.76	3.29/2.26	0	8	0.35	−0.80
SPQ ideas of reference	0.74	3.10/2.33	0	9	0.44	−0.64

*n* = 1,156 unless otherwise noted. TMMS, Trait Meta-Mood Scale; Ment-S, Mentalization Scale; CTQ, Childhood Trauma Questionnaire; CECA.Q, Childhood Care and Abuse Questionnaire; CAPE, Community Assessment of Psychic Experiences; MSS, Multidimentsional Schizotypy Scale; SPQ, Schizotypal Personality Questionnaire.

a Note that this subscale includes four items and parental losses do not necessarily covary, so internal consistency is expected to be low.

b See the methods section for explanation of lower n for these variables.

c These are the means of standardized z-scores of the subscales for psychological abuse (α = 0.82, 0.78 for father and mother, respectively) and antipathy (α = 0.74, 0.81 for father and mother, respectively).

To reduce factors for childhood trauma (to 5 predictor variables) and the number of mediation models, two *a priori* analyses were conducted using trauma measures. Given the low-endorsement rates of sexual abuse and that primarily modest-to-high correlations (0.26–0.65) were observed between CTQ non-sexual abuse subscales (emotional abuse, emotional neglect, physical abuse, and physical neglect), and following Sheinbaum et al. (86), principal components analysis (PCA) was conducted to produce a single emotional/physical adversity factor. This PCA yielded one component that explained 59% of the variance. Additionally, due to high correlations (*r* = 0.75 for father and *r* = 0.76 for mother, *p* < 0.001 for both) rather than exploring CECA.Q psychological abuse and CECA.Q antipathy separately for maternal and paternal figures, we elected to combine standardized (z) scores for available data on psychological abuse and antipathy into one measure of adversity for each parent: CECA.Q maternal abuse and CECA.Q paternal abuse.

**Figure 1:**
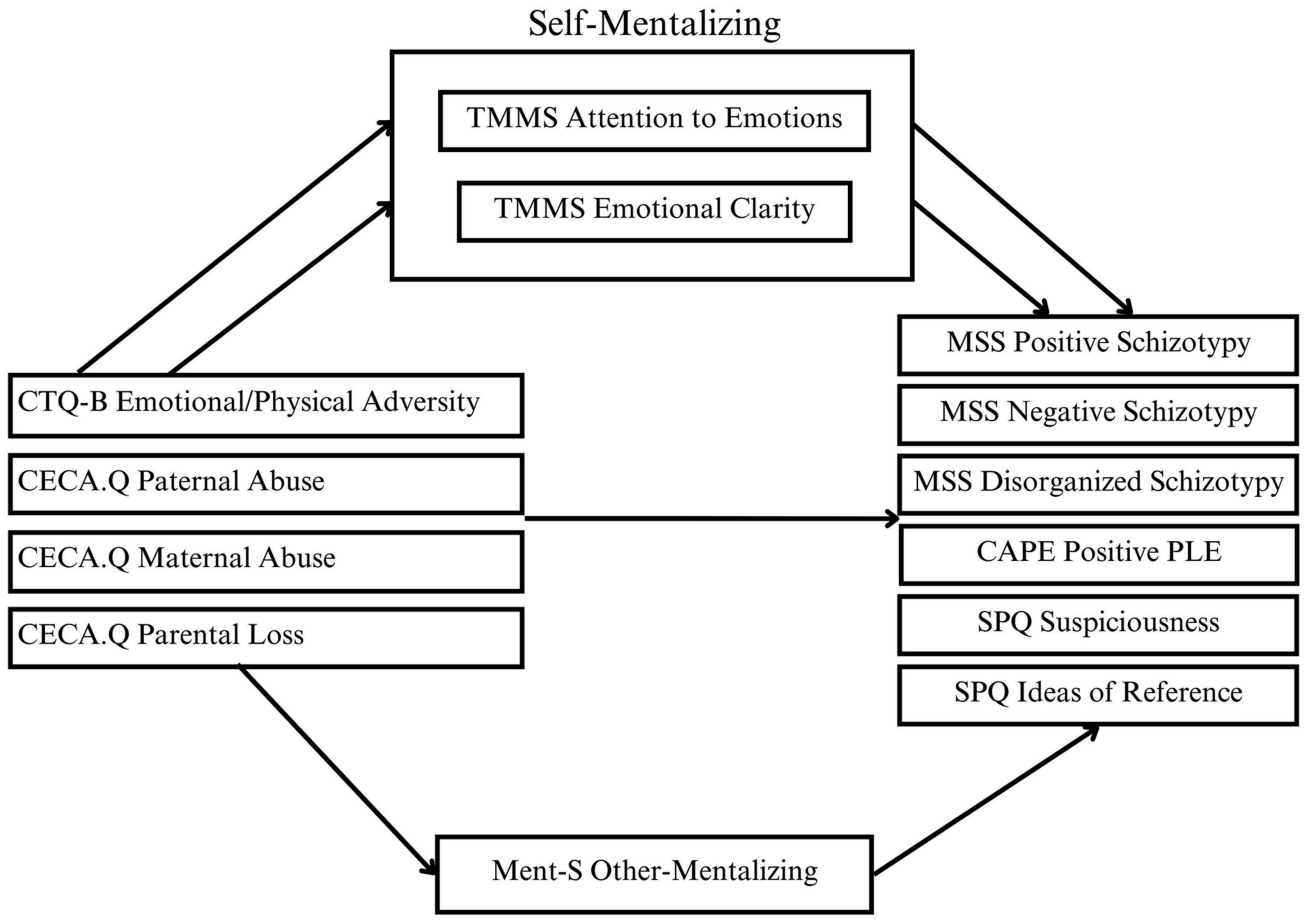
Parallel multiple mediation models evaluating mediation of mentalizing factors in the relationships between childhood adversity and psychotic-like outcomes.

Moderate-strong correlations (0.47–0.57) were observed between mentalization domains, and thus the three mentalizing factors—attention to emotions, emotional clarity and other-mentalizing—were simultaneously entered into parallel multiple mediation models for all outcome variables. A visual depiction of the *a-priori* mediation models tested can be seen in Figure 1. Parallel multiple mediation analyses were conducted using Hayes (87) PROCESS Macro Model 4 for assessing indirect pathways. Compared to the use of several single mediation analyses, parallel mediation accounts for the variance of other mediators in the model and is well-suited to inter-correlated mediators, as it offers a more precise estimate of indirect effects. This technique has been repeatedly demonstrated as useful in psychosis research (86, 88, 89).

Mediations of the associations of trauma and psychosis outcomes via mentalization are demonstrated by significant indirect coefficients with lower-and upper-bound confidence intervals that do not include zero. Six mediation models were tested for each of the nonclinical psychosis outcomes (positive, negative and disorganized schizotypy, positive PLE, suspiciousness, and ideas of reference) and with each of the five trauma indicators (emotional/physical adversity, maternal abuse, paternal abuse, role reversal, and parental loss) as the independent variable and the three mentalizing domains (attention to emotions, emotional clarity, and other-mentalizing) entered simultaneously as mediators, resulting in a total of 30 mediation models analyzed. Analyses were limited to these *a priori* hypothesized models. Bootstrapping with 10,000 resamples was conducted to generate bias-corrected confidence intervals.

## 3 Results

Descriptive statistics for all variables are presented in Table 1 and Pearson correlations are in Table 2. Despite small effect sizes, direction of associations as displayed in Table 2 indicates a pattern of positive associations for attention to emotions with schizotypy, PLE and suspiciousness, but negative associations with emotional clarity. Meanwhile, other-mentalizing was had small to moderate sized associations with negative schizotypy and disorganized schizotypy, but was not associated with positive schizotypy, PLE or paranoia. Moderate or near-moderate associations were observed for negative schizotypy with all mentalization factors and for disorganized schizotypy with emotional clarity.

**Table 2:** Pearson correlations for study variables (*n* = 1,156).

	TMMS clarity	Ment-S other-mentalizing	CTQ emotional/physical adversity	CECA.Q maternal abuse	CECA.Q paternal abuse	CECA.Q parental loss	CECA.Q role reversal	MSS positive schizotypy	MSS negative schizotypy	MSS disorganized schizotypy	CAPE positive PLE	SPQ suspiciousness	SPQ ideas of reference
TMMS attention	0.53***	0.47***	0.08*	0.06*	0.12***	0.03	0.08**	0.16***	−0.26***	0.02	0.13***	0.10**	0.14***
TMMS clarity		0.56***	−0.07*	−0.07*	−0.03	−0.01	0.01	−0.06	−0.26***	−0.37***	−0.13***	−0.19***	−0.17***
Ment-S other-mentalizing			−0.00	0.05	0.03	0.03	0.12**	0.07*	−0.29***	−0.17***	0.01	−0.04	−0.01
CTQ emotional/physical adversity				0.56***	0.57***	0.23***	0.41**	0.20***	0.22***	0.31***	0.24***	0.29***	0.16***
CECA.Q maternal abuse					0.35***	0.08**	0.34**	0.21***	0.11***	0.29***	0.23***	0.26***	0.20***
CECA.Q paternal abuse						0.10**	0.24**	0.14***	0.09**	0.27***	0.22***	0.24***	0.17***
CECA.Q parental loss							0.24**	−0.01	0.08**	0.06	0.03	0.06*	−0.01
CECA.Q role reversal								0.19**	0.08**	0.17**	0.18**	0.16**	0.13**
MSS positive schizotypy									0.10**	0.38***	0.74***	0.44***	0.59***
MSS negative schizotypy										0.31***	0.18***	0.22***	0.11***
MSS disorganized schizotypy											0.48***	0.54***	0.43***
CAPE positive PLE												0.56***	0.65***
SPQ suspiciousness													0.57***

*p < 0.05; **p < 0.01; ***p < 0.001. Medium effect sizes in bold, large effect sizes in bold and italics. TMMS, Trait Meta-Mood Scale; Ment-S, Mentalization Scale; CTQ, Childhood Trauma Questionnaire; CECA.Q, Childhood Care and Abuse Questionnaire; MSS, Multidimentsional Schizotypy Scale; CAPE, Community Assessment of Psychic Experiences; SPQ, Schizotypal Personality Questionnaire.

### 3.1 Mediation analyses

After entering the three mentalization factors as parallel mediators in the models, increased attention to emotions was a significant partial mediator for most models including intentional forms of adversity (i.e., emotional/physical adversity, maternal and paternal abuse, and role reversal) and decreased emotional clarity for some models. All significant mediations were partial. Note that the general direction of effects for attention to emotions and emotional clarity in the mediation models is consistent with correlational analysis; that is, higher scores for attention to emotions is associated with higher outcomes, while lower clarity is associated with higher outcomes. Other-mentalizing was only a significant mediator in one model that examined the relationship between role reversal and negative schizotypy.

For the model using the combined emotional/physical adversity component, parameter estimates of the direct, total, and indirect effects can be found in Table 3. Indirect effects for increased attention to emotions in this model were significant for positive schizotypy, negative schizotypy, disorganized schizotypy, PLE, suspiciousness and ideas of reference. There was a significant indirect effect for emotional/physical adversity via emotional clarity on positive schizotypy, PLE and suspiciousness. Indirect effects of emotional clarity on positive schizotypy, disorganized schizotypy and positive PLE were such that trauma was associated with lower emotional clarity which is, in turn, associated with higher scores on psychotic outcomes; however, the effect of emotional/physical adversity on suspiciousness via emotional clarity was the opposite, such that lower emotional clarity was related to decreased suspiciousness. Outcomes that were significant for both attention to emotions and emotional clarity in this model reflect relatively equivalent effect sizes for the two specific indirect effects, with the exception of disorganized schizotypy which was driven predominantly by emotional clarity.

**Table 3:** Parallel multiple mediation analyses examining indirect effects of CTQ motional/physical adversity on nonclinical psychotic phenomena via self-mentalizing factors (1) attention to emotions and (2) clarity of emotions, and (3) other-mentalizing.

	Unstandardized parameter estimate	SE	95% Bias-corrected confidence interval	
			Lower	Upper
Positive schizotypy				
Total effect	0.5357^*^	0.0772	0.3842	0.6872
Direct effect	0.4511^*^	0.0763	0.3014	0.6008
Indirect total effect	0.0846^*^	0.0201	0.0477	0.1268
Indirect effect via attention to emotions	0.0456^*^	0.0203	0.0086	0.0879
Indirect effect via emotional clarity	0.0393^*^	0.0210	0.0002	0.0834
Indirect effect via other-mentalizing	−0.0004	0.0077	−0.0159	0.0154
Negative schizotypy				
Total effect	0.4678^*^	0.0618	0.3466	0.5889
Direct effect	0.4866^*^	0.0588	0.3712	0.6021
Indirect total effect	−0.0189	0.0255	−0.0682	0.0324
Indirect effect via attention to emotions	−0.0274^*^	0.0130	−0.0559	−0.0047
Indirect effect via emotional clarity	0.0079	0.0077	−0.0041	0.0256
Indirect effect via other-mentalizing	0.0006	0.0118	−0.0223	0.0252
Disorganized schizotypy				
Total effect	1.1175^*^	0.1016	0.9181	1.3169
Direct effect	0.9270^*^	0.0927	0.7451	1.1089
Indirect total effect	0.1905	0.0492	0.0957	0.2871
Indirect effect via attention to emotions	0.0752	0.0329	0.0128	0.1424
Indirect effect via emotional clarity	0.1146	0.0598	−0.0036	0.2304
Indirect effect via other-mentalizing	0.0008	0.0057	−0.0102	0.0151
Positive PLE				
Total effect	1.5530^*^	0.1858	1.1885	1.9175
Direct effect	1.3257^*^	0.1824	0.9679	1.6835
Indirect total effect	0.2273^*^	0.0526	0.1308	0.3369
Indirect effect via attention to emotions	0.1069^*^	0.0501	0.0136	0.2112
Indirect effect via emotional clarity	0.1242^*^	0.0621	0.0043	0.2498
Indirect effect via other-mentalizing	−0.0038	0.0137	−0.0344	0.0236
Suspiciousness				
Total effect	0.6535^*^	0.0638	0.5283	0.7787
Direct effect	0.5575^*^	0.0618	0.4363	0.6786
Indirect total effect	0.0960^*^	0.0203	0.0576	0.1381
Indirect effect via attention to emotions	0.0437^*^	0.0187	0.0079	0.0820
Indirect effect via emotional clarity	0.0524^*^	0.0264	0.0012	0.1050
Indirect effect via other-mentalizing	−0.0001	0.0034	−0.0073	0.0077
Ideas of reference				
Total effect	0.3628^*^	0.0678	0.2297	0.4958
Direct effect	0.2520^*^	0.0652	0.1240	0.3800
Indirect total effect	0.1108^*^	0.0231	0.0667	0.1574
Indirect effect via attention to emotions	0.0550^*^	0.0237	0.0100	0.1026
Indirect effect via emotional clarity	0.0559	0.0290	−0.0012	0.1136
Indirect effect via other-mentalizing	−0.0002	0.0041	−0.0090	0.0084

^*^95% CI does not include zero.

In the model for paternal abuse (Table 4), there was a significant indirect effect of paternal abuse on all outcomes via higher attention to emotions. In the case of negative schizotypy, the indirect total effect of all mediators combined was nonsignificant, indicating that there was no parallel mediation, but that attention to emotions remained a significant mediator of the association between paternal abuse and negative schizotypy after controlling for the other mediators (emotional clarity and other-mentalizing). Unlike other outcomes, the indirect effect of increased attention to emotions for this model was related to lower scores of negative schizotypy. No significant indirect effects were found for emotional clarity or other-mentalizing in the relationships between paternal abuse and psychosis outcomes. Mediating effects of mentalization on all outcomes had relatively small effect sizes but were most pronounced for positive PLE and disorganized schizotypy, which are roughly double those of other outcomes.

**Table 4:** Parallel multiple mediation analyses examining indirect effects of CECA.Q paternal abuse on nonclinical psychotic phenomena via self-mentalizing factors (1) attention to emotions and (2) clarity of emotions, and (3) other-mentalizing.

	Unstandardized parameter estimate	SE	95% Bias-corrected confidence interval	
			Lower	Upper
Positive schizotypy				
Total effect	0.3868^*^	0.0877	0.2148	0.5588
Direct effect	0.2936^*^	0.0868	0.1232	0.4640
Indirect total effect	0.0932^*^	0.0229	0.0522	0.1414
Indirect effect via attention to emotions	0.0743^*^	0.0245	0.0308	0.1265
Indirect effect via emotional clarity	0.0148	0.0211	−0.0272	0.0585
Indirect effect via other-mentalizing	0.0041	0.0064	−0.0068	0.0192
Negative schizotypy				
Total effect	0.2122^*^	0.0714	0.0721	0.3524
Direct effect	0.2609^*^	0.0680	0.1275	0.3942
Indirect total effect	−0.0486	0.0292	−0.1062	0.0079
Indirect effect via attention to emotions	−0.0417^*^	0.0166	−0.0776	−0.0135
Indirect effect via emotional clarity	0.0041	0.0071	−0.0080	0.0208
Indirect effect via other-mentalizing	−0.0110	0.0135	−0.0391	0.0150
Disorganized schizotypy				
Total effect	1.0229^*^	0.1173	0.7928	1.2530
Direct effect	0.8631^*^	0.1062	0.6547	1.0715
Indirect total effect	0.1598^*^	0.0570	0.0488	0.2735
Indirect effect via attention to emotions	0.1327^*^	0.0385	0.0612	0.2123
Indirect effect via emotional clarity	0.0318	0.0661	−0.0984	0.1646
Indirect effect via other-mentalizing	−0.0047	0.0083	−0.0235	0.0107
Positive PLE				
Total effect	1.4384^*^	0.2106	1.0250	1.8518
Direct effect	1.2034^*^	0.2069	0.7973	1.6095
Indirect total effect	0.2350^*^	0.0630	0.1200	0.3668
Indirect effect via attention to emotions	0.1954^*^	0.0615	0.0883	0.3274
Indirect effect via emotional clarity	0.0333	0.0687	−0.1048	0.1685
Indirect effect via other-mentalizing	0.0063	0.0137	−0.0166	0.0406
Suspiciousness				
Total effect	0.5659^*^	0.0738	0.4210	0.7107
Direct effect	0.4743^*^	0.0715	0.3340	0.6147
Indirect total effect	0.0915^*^	0.0239	0.0457	0.1399
Indirect effect via attention to emotions	0.0682^*^	0.0220	0.0289	0.1146
Indirect effect via emotional clarity	0.0210	0.0291	−0.0364	0.0781
Indirect effect via other-mentalizing	0.0024	0.0045	−0.0049	0.0134
Ideas of reference				
Total effect	0.4308^*^	0.0783	0.2772	0.5844
Direct effect	0.3154^*^	0.0752	0.1679	0.4629
Indirect total effect	0.1154^*^	0.0278	0.0626	0.1725
Indirect effect via attention to emotions	0.0899^*^	0.0273	0.0159	0.0583
Indirect effect via emotional clarity	0.0229	0.0318	−0.0392	0.0853
Indirect effect via other-mentalizing	0.0026	0.0047	−0.0051	0.0142

^*^95% CI does not include zero.

The model evaluating the multiple parallel mediation model between maternal abuse and psychosis outcomes with mentalization factors as mediators is presented in Table 5. There was a significant indirect effect of maternal abuse on psychosis outcomes via greater attention to emotions for positive schizotypy, disorganized schizotypy, PLE, suspiciousness and ideas of reference. In the case of negative schizotypy, maternal abuse was associated with higher attention, but decreased negative schizotypy. Contrary to results for paternal abuse, significant indirect effects of decreased emotional clarity were also found for maternal abuse on most outcomes: positive schizotypy, disorganized schizotypy, suspiciousness and ideas of reference. All outcomes had a significant indirect total effect (Table 5) indicating multiple parallel mediation, except for negative schizotypy, which indicates that its only significant mediator, attention to emotions, has an indirect effect on negative schizotypy even after controlling for effects of other mediators. Although emotional clarity did not reach significance as a mediator for paternal abuse, in general, the effect sizes for specific indirect effects of attention to emotions between maternal abuse and outcomes are roughly half of those for paternal abuse.

**Table 5:** Parallel multiple mediation analyses examining indirect effects of standardized scores of CECA.Q maternal abuse on nonclinical psychotic phenomena via self-mentalizing factors (1) attention to emotions and (2) clarity of emotions, and (3) other-mentalizing.

	Unstandardized parameter estimate	SE	95% Bias-corrected confidence interval	
			Lower	Upper
Positive schizotypy				
Total effect	0.5849^*^	0838	0.4205	0.7493
Direct effect	0.4934^*^	0.0831	0.3303	0.6565
Indirect total effect	0.0915^*^	0.0233	0.0494	0.1410
Indirect effect via attention to emotions	0.0390^*^	0.0204	0.0018	0.0823
Indirect effect via emotional clarity	0.0422^*^	0.0198	0.0058	0.0838
Indirect effect via other-mentalizing	0.0104	0.0090	−0.0022	0.0317
Negative schizotypy				
Total effect	0.2511^*^	0.0676	0.1184	0.3837
Direct effect	0.2818^*^	0.0645	0.1552	0.4085
Indirect total effect	−0.0308	0.0272	−0.0847	0.0220
Indirect effect via attention to emotions	−0.0220^*^	0.0124	−0.0491	−0.0008
Indirect effect via emotional clarity	0.0116	0.0091	−0.0020	0.0332
Indirect effect via other-mentalizing	−0.0204	0.0138	−0.0489	0.0049
Disorganized schizotypy				
Total effect	1.1017^*^	0.1105	0.8849	1.3184
Direct effect	0.9122^*^	0.1004	0.7151	1.1092
Indirect total effect	0.1895^*^	0.0565	0.0809	0.3017
Indirect effect via attention to emotions	0.0752^*^	0.0350	0.0086	0.1470
Indirect effect via emotional clarity	0.1245^*^	0.0601	0.0096	0.2439
Indirect effect via other-mentalizing	−0.0102	0.0095	−0.0323	0.0046
Positive PLE				
Total effect	1.5109^*^	0.2007	1.1172	1.9047
Direct effect	1.2644^*^	0.1977	0.8765	1.6523
Indirect total effect	0.2465^*^	0.0613	0.1348	0.3746
Indirect effect via attention to emotions	0.1020^*^	0.0513	0.0069	0.2098
Indirect effect via emotional clarity	0.1281^*^	0.0610	0.0142	0.2530
Indirect effect via other-mentalizing	0.0164	0.0181	−0.0093	0.0617
Suspiciousness				
Total effect	0.6373^*^	0.0700	0.4999	0.7747
Direct effect	0.5377^*^	0.0680	0.4043	0.6712
Indirect total effect	0.0996^*^	0.0236	0.0557	0.1477
Indirect effect via attention to emotions	0.0389^*^	0.0199	0.0012	0.0797
Indirect effect via emotional clarity	0.0581^*^	0.0259	0.0082	0.1099
Indirect effect via other-mentalizing	0.0026	0.0052	−0.0057	0.0155
Ideas of reference				
Total effect	0.4916^*^	0.0736	0.3471	0.6361
Direct effect	0.3761^*^	0.0711	0.2366	0.5157
Indirect total effect	0.1155^*^	0.0266	0.0646	0.1686
Indirect effect via attention to emotions	0.0476^*^	0.0238	0.0019	0.0961
Indirect effect via emotional clarity	0.0624^*^	0.0281	0.0074	0.1194
Indirect effect via other-mentalizing	0.0054	0.0062	−0.0013	0.0082

^*^95% CI does not include zero.

There was a significant indirect effect of role reversal on all indicators of schizotypy, positive PLE and paranoia via attention to emotions, which were most pronounced for the positive dimension (Table 6). Significant indirect effects were found via emotional clarity for suspiciousness, positive schizotypy, and ideas of reference. The only significant indirect effect of other-mentalizing was found in this model for the association between role reversal and negative schizotypy. Effect sizes in this model are attenuated compared to other models.

**Table 6:** Parallel multiple mediation analyses examining indirect effects of CECA.Q Role Reversal on nonclinical psychotic phenomena via self-mentalizing factors (1) attention to emotions and (2) clarity of emotions, and (3) other-mentalizing.

	Unstandardized parameter estimate (value of *p*)	SE	95% Bias-corrected confidence interval	
			Lower	Upper
Positive schizotypy				
Total effect	0.0719^*^	0.0109	0.0505	0.0933
Direct effect	0.0630^*^	0.0108	0.0419	0.0841
Indirect total effect	0.0088^*^	0.0031	0.0030	0.0152
Indirect effect via attention to emotions	0.0069^*^	0.0029	0.0015	0.0130
Indirect effect via emotional clarity	−0.0010^*^	0.0027	−0.0063	0.0042
Indirect effect via other-mentalizing	0.0029	0.0018	−0.0002	0.0068
Negative schizotypy				
Total effect	0.0244^*^	0.0089	0.0070	0.0419
Direct effect	0.0350^*^	0.0085	0.0183	0.0516
Indirect total effect	−0.0105^*^	0.0035	−0.0176	−0.0039
Indirect effect via attention to emotions	−0.0032^*^	0.0016	−0.0067	−0.0006
Indirect effect via emotional clarity	−0.0003	0.0009	−0.0022	0.0014
Indirect effect via other-mentalizing	−0.0071^*^	0.0024	−0.0123	−0.0029
Disorganized schizotypy				
Total effect	0.0865^*^	0.0148	0.0574	0.1155
Direct effect	0.0805^*^	0.0134	0.0542	0.1067
Indirect total effect	0.0060	0.0076	−0.0090	0.0209
Indirect effect via attention to emotions	0.0114^*^	0.0048	0.0020	0.0212
Indirect effect via emotional clarity	−0.0016	0.0080	−0.0172	0.0143
Indirect effect via other-mentalizing	−0.0038	0.0024	−0.0092	0.0003
Positive psychotic-like experiences				
Total effect	0.1635^*^	0.0265	0.1115	0.2155
Direct effect	0.1438^*^	0.0259	0.0930	0.1947
Indirect total effect	0.0196^*^	0.0084	0.0038	0.0364
Indirect effect via attention to emotions	0.0162^*^	0.0073	0.0028	0.0312
Indirect effect via emotional clarity	−0.0001	0.0081	−0.0161	0.0158
Indirect effect via other-mentalizing	0.0035	0.0040	−0.0039	0.0121
Suspiciousness				
Total effect	0.0521^*^	0.0093	0.0339	0.0703
Direct effect	0.0458^*^	0.0089	0.0283	0.0633
Indirect total effect	0.0063^*^	0.0034	−0.0005	0.0131
Indirect effect via attention to emotions	0.0069^*^	0.0029	0.0015	0.0128
Indirect effect via emotional clarity	−0.0013^*^	0.0036	−0.0086	0.0057
Indirect effect via other-mentalizing	0.0007	0.0014	−0.0021	0.0037
Ideas of reference				
Total effect	0.0424^*^	0.0096	0.0235	0.0612
Direct effect	0.0344^*^	0.0092	0.0164	0.0525
Indirect total effect	0.0079^*^	0.0037	0.0007	0.0152
Indirect effect via attention to emotions	0.0080^*^	0.0032	0.0019	0.0144
Indirect effect via emotional clarity	−0.0014	0.0037	−0.0087	0.0059
Indirect effect via other-mentalizing	0.0013	0.0015	−0.0015	0.0044

^*^95% CI does not include zero.

Parental loss was not related to any psychosis outcomes via mentalizing (Table 7). The only significant results were found for negative schizotypy and suspiciousness. There was a significant effect of parental loss on negative schizotypy after controlling for all mediators and a significant total effect of parental loss on negative schizotypy, along with a significant total effect of parental loss on suspiciousness.

**Table 7:** Parallel multiple mediation analyses examining indirect effects of CECA.Q parental loss on nonclinical psychotic phenomena via self-mentalizing factors (1) attention to emotions and (2) clarity of emotions, and (3) other-mentalizing.

	Unstandardized parameter estimate (value of *p*)	SE	95% Bias-corrected confidence interval	
			Lower	Upper
Positive schizotypy				
Total effect	−0.0551	0.1609	−0.3708	0.2606
Direct effect	−0.1231	0.1564	−0.4300	0.1839
Indirect total effect	0.0680	0.0362	−0.0008	0.1407
Indirect effect via attention to emotions	0.0346	0.0390	−0.0421	0.1125
Indirect effect via emotional clarity	0.0183	0.0410	−0.0612	0.1019
Indirect effect via other-mentalizing	0.0150	0.0167	−0.0151	0.0528
Negative schizotypy				
Total effect	0.3594^*^	0.1287	0.1068	0.6120
Direct effect	0.3929^*^	0.1218	0.1538	0.6319
Indirect total effect	−0.0335	0.0448	−0.1204	0.0556
Indirect effect via attention to emotions	−0.0149	0.0175	−0.0516	0.0184
Indirect effect via emotional clarity	0.0051	0.0126	−0.0183	0.0341
Indirect effect via other-mentalizing	−0.0238	0.0244	−0.0750	0.0216
Disorganized schizotypy				
Total effect	0.4118	0.2181	−0.0161	0.8398
Direct effect	0.3161	0.1955	−0.0674	0.6966
Indirect total effect	0.0957	0.0961	−0.0883	0.2845
Indirect effect via attention to emotions	0.0425	0.0656	−0.0887	0.1738
Indirect effect via emotional clarity	0.0631	0.1160	−0.1624	0.2951
Indirect effect via other-mentalizing	−0.0099	0.0142	−0.0433	0.0126
Positive psychotic-like experiences				
Total effect	0.3829	0.3903	−0.3829	1.1487
Direct effect	0.2316	0.3768	−0.5078	0.9709
Indirect total effect	0.1513	0.0967	−0.0352	0.3425
Indirect effect via attention to emotions	0.0616	0.0998	−0.1322	0.2598
Indirect effect via emotional clarity	0.0650	0.1229	−0.1761	0.3087
Indirect effect via other-mentalizing	0.0247	0.0313	−0.0246	0.1018
Suspiciousness				
Total effect	0.2798^*^	0.1358	0.0133	0.5464
Direct effect	0.2167	0.1290	−0.0364	0.4698
Indirect total effect	0.0632	0.0409	−0.0158	0.1456
Indirect effect via attention to emotions	0.0399	0.0384	−0.0408	0.1102
Indirect effect via emotional clarity	0.0239	0.0531	−0.0815	0.1305
Indirect effect via other-mentalizing	0.0054	0.0087	−0.0077	0.0279
Ideas of Reference				
Total effect	−0.0487	0.1402	−0.3237	0.2262
Direct effect	−0.1194	0.1327	−0.3797	0.1409
Indirect total effect	0.0706	0.0421	−0.0110	0.1542
Indirect effect via attention to emotions	0.0394	0.0437	−0.0476	0.1236
Indirect effect via emotional clarity	0.0242	0.0536	−0.0799	0.1328
Indirect effect via other-mentalizing	0.0071	0.0101	−0.0080	0.0067

^*^95% CI does not include zero.

## 4 Discussion

The present study explored, for the first time, the relationship between the self and other dimensions of mentalization with schizotypy, and extended these findings by examining the mediating role of self- and other-mentalization in the associations between a wide range of childhood adversities, including intentional (i.e., emotional/physical adversity, maternal and paternal abuse, and role reversal) and nonintentional (i.e., parental loss) harm, and psychotic-like outcomes.

Overall, associations of mentalizing domains with the schizotypy dimensions were consistent with previous research in other mental health phenotypes, such that attention to emotions is positively associated with impairment and increased symptoms, while emotional clarity is consistently supported as a protective factor, or, in other words, one that attenuates impairment (61, 62, 90, 91). The positive schizotypy dimension was directly and more strongly correlated with attention to emotions than with emotional clarity, which is consistent with findings that positive schizotypy is associated with increased attention to emotions in general, lower clarity (90) and lower emotional recognition (92). Interestingly, the disorganized dimension had a nonsignificant correlation with attention to emotions, but a moderate inverse association with emotional clarity, suggesting that independent of how much people with disorganized schizotypy attend to their thoughts, they struggle to understand them and thus, lack clarity. One study found that clarity of self-concept is more transient in clinical psychosis and demonstrated that decreased clarity was associated with both positive and negative psychosis symptoms, however disorganized symptoms were not evaluated (63). Recent studies have suggested that emotional dysregulation is a core component of the disorganized schizotypy dimension (93).

Overall, a pattern of significant parallel multiple mediation was observed for all models including intentional, but not nonintentional, forms of adversity and all psychotic-like traits and experiences except negative schizotypy. Specific indirect effects revealed that childhood adversity is related to increased levels of psychotic-like features through increased attention and secondarily through decreased clarity, but that other-mentalizing is not a relevant factor in these relationships. As the singular exception, the model examining the impact of role reversal showed a significant parallel multiple mediating effect on almost all psychotic-like features, including negative but not disorganized schizotypy, and the association between role reversal and negative schizotypy was significantly mediated by other-mentalizing. Decreased emotional clarity and increased attention to emotions were significant mediators in the associations between maternal abuse and nearly all psychosis spectrum outcomes (except negative schizotypy), whereas for paternal abuse, significant indirect effects were only found for attention to emotions. There was no significant indirect effect of parental loss on psychotic-like features via mentalizing.

Most of the parallel mediation models were not significant for negative schizotypy; however, specific indirect effects suggest that increased attention to emotions was inversely associated with negative schizotypy, consistent with the well-established finding that negative schizotypy is associated with diminished emotional expression and experience (32, 94). This dimension is characterized by alogia, anergia, avolition, anhedonia, flat affect, and a general disinterest in other people and the world as a whole (27). In general, comparisons with antecedent, similar research are difficult to make as only one other study has evaluated such relationships (albeit in a clinical sample) (25), however the models are incomparable as the previous paper followed mediation requirements outlined by Baron & Kenny (95), which supposes that all variables must be associated to conduct mediation and thus indirect effects were only evaluated (and supported) for negative symptoms. Conversely, our study followed the process outlined by Hayes (96), which does not require empirical associations, but instead, theoretical support for proposed indirect associations. Thus, several additional models were conducted that revealed, we believe, thought-provoking results.

### 4.1 Intentional versus nonintentional childhood adversity

To our knowledge, no previous research has evaluated whether there are differential effects of mentalization on the relationship between intentional versus general, unintentional childhood adversity and psychotic outcomes, and particularly not in a nonclinically ascertained sample. Results revealed that only intentionally harmful childhood adversity (i.e., maltreatment and neglect) impacted mentalization functioning, compared to nonintentional childhood adversity as indexed by parental loss. The measure used for parental loss in this study assesses the loss of one or both parent figures before age 18 due to death, separation, or abandonment. Such losses are certainly impactful to those who suffer them, as they almost inevitably result in a pivotal destabilization of family, extensive emotional consequences, and often essential and monumental family restructuring (97). Despite the repercussions of a central loss such as that of a parent figure, no significant effects were found on psychotic-like features via mentalizing, and parental loss did not affect most outcomes, even after controlling for mentalization levels. This could indicate that mentalization is negatively affected more by central but harmful attachment figures than potential consequences to attachment relationships following parental loss such as (1) a lack of attachment figures, or (2) more ‘distant’ attachment figures, perhaps outside of the family, that develop epistemic trust with a child after they suffer parental loss. Indeed, some literature suggests that adjustment difficulties following bereavement are not consistently related to grieving but are instead accounted for by inadequate care following parental loss (98). Such a finding emphasizes the importance of a parent’s role as a supportive, understanding, and responsible adult figure, rather than a dangerous and untrustworthy one (99).

### 4.2 Differential effects of maternal and paternal abuse

A non-hypothesized finding that merits further study was the differential effects of maternal versus paternal abuse on self-mentalizing factors. Results suggest that increased attention in the wake of childhood adversity is more impactful in the case of paternal abuse, as coefficients for the mediating role of attention to emotions are roughly double the same coefficients for maternal abuse for all outcomes. Nonetheless, in the case of maternal abuse the additional mediating effect of impaired clarity is present for all outcomes excepting negative schizotypy. Despite gender-role stereotypes whereby maternal figures are responsible for child rearing and paternal figures provide resources for the family outside the home being challenged in recent years, the mean age of our sample suggests that most maternal figures may still be the principal caregiver. Considering that mentalization is usually developed through relationships with said primary caregiver(s) (9), mentalization could be severely impacted if the mother–child relationship is damaged. For example, a ‘good enough mother’ conceptualized by Winnicott (100) and later expanded upon through attachment relationships by Bowlby (101) is suggested to be necessary for adequate child development, particularly of socioemotional abilities such as mentalization. If, for example, the maternal figure is the primary caregiver, but instead of providing security, stability and fostering epistemic trust with the child, fails to play this role and breaks epistemic trust by engaging in abusive or neglectful behavior, the child’s mentalization skills may be more highly impaired than they would in a father-child relationship. This notion is supported in a recent study which demonstrates that maternal psychological states are more impactful on children’s adult clinical psychosis status than paternal psychological states (102). In cases where the father is the primary caregiver, perhaps this dynamic could be expected to be reversed, however, extant literature does not shed light on this question.

### 4.3 Know thyself: the role of the self

An overwhelming pattern of significant findings for self- but not other-mentalizing was revealed in analyses of this study. Extant literature suggests that adaptive emotional strategies are helpful in preventing psychosis, and that emotional clarity has been shown to be ‘protective’ from the development of other mental health disorders (103). This, combined with evidence of (especially self-) mentalization as a transdiagnostic protective factor (8, 60, 62, 91), suggests that maintaining good self-mentalizing in the wake of adverse events could potentially result in better outcomes. The implication of an impaired understanding of the self in psychosis is well-accepted, with results suggesting that disturbances in understanding and identifying with the self may underpin self-disorders which hyper-aggregate in psychotic spectrum disorders (104, 105). Perhaps the ability to self-mentalize, developed during the formative years, could impede significant impairments in the development of self-identity, protecting from psychotic outcomes. Although the precise role of self-mentalization is not well understood, results that partially support our findings have been found in various stages of the psychosis spectrum; for example in self-concept clarity (63), misattributions of self-referential representations (106, 107), and even after traumatic life events which importantly interact with the self to affect psychosis proneness (108). Evidence supporting good mentalizing as a buffer for the impact of persecutory delusions (positive dimension) on functioning (109) further suggests that mentalizing can be protective, but, when impaired conveys risk.

Until the call for evaluation of distinct mentalization polarities (59), mentalization was evaluated solely as a general construct, without exploring differential contributions of self- vs. other- polarities of mentalization. Nonetheless, positive symptoms are highly implicated in self-identity and self-boundary (53, 54) and thus measures of self-mentalization may more precisely capture characteristics of positive schizotypy than other-mentalization. Indeed, contemporary cognition research suggests that understanding of the ‘self’ forms the stem of understanding the ‘other’ (110). Overall, this evidence combined with the consideration that psychotic symptoms are viewed as a ‘disturbance to the self’, and that self-mentalizing gives rise to self-organization, emotional regulation, and sense of agency, might account for the fact that that psychosis spectrum impairments are substantially associated with self-mentalizing. Of note, self-mentalizing not only impacted positive psychotic-like features, but also the disorganized schizotypy dimension. Potentially, impaired self-mentalizing (i.e., increased attention but decreased clarity, in alignment with our results) after the exposure to childhood adversity negatively impacts the ability to organize and express thoughts and behavior, that is, resulting in a manifestation of disorganized schizotypal features.

Overall, our lack of significant findings for other-mentalizing could be due to higher discrimination of the mentalization construct in the current study, whereby self- and other-mentalizing are separated, revealing that self-mentalizing drives associations. In fact, only one model revealed an indirect effect via other-mentalizing, in which childhood experiences of role reversal decreased negative schizotypy through increased other-mentalizing. To date, mentalization has been operationalized primarily using various Theory of Mind tasks, which overall evaluate other-mentalizing. These studies support (other-) mentalizing as a mediator of childhood neglect and psychosis symptoms (47), of trauma/expressed emotion and schizotypal symptoms (72) and have even found that (other-) mentalizing fully mediates the association between social perception difficulties and negative symptoms (71). Although role reversal and its impact on other-mentalizing has not been examined previously, one may speculate that assuming parental responsibilities and providing emotional support to the parent from a young age might subserve the development of an increased capacity to think about and understand other’s emotions and needs (i.e., other-mentalizing) and this, in turn, may increase one’s curiosity and openness to the world (i.e., diminished negative schizotypy).

### 4.4 Strengths and limitations

This study benefits from (1) its novelty in exploring self- and other-mentalization in a combined study, (2) exploring a wide range of childhood adversity types, including distinctions between intentional and nonintentional harm, (3) a unique examination of the role of maternal versus paternal abuse, and (4) the assessment of psychosis spectrum outcomes in an extensive sample of nonclinical young adults. While assessing impairment at the clinical level is helpful, it may not be early enough to develop interventions and understanding that can ultimately prevent severe functional impairment, particularly in the case of psychosis (110). Schizotypy offers a unifying construct for the psychosis spectrum that provides benefits for understanding the role of mechanisms such as mentalization in the development of disorders (26, 35). More so, acknowledging the multidimensionality of the construct allows to unravel the distinct etiological and developmental pathways that specifically lead to positive, negative or disorganized manifestations (27). Thus, the study of schizotypy features in a nonclinically ascertained sample may, in fact, be a chief strength of the study.

Unfortunately, the cross-sectional nature of our study design does not allow for causal associations to be evaluated, although hypotheses were made based on extant literature and theoretical grounding which guided subsequent analysis; thus, the present findings should be replicated in longitudinal studies. A small amount of missing data due an error in survey administration software resulted in few items being removed from certain measures for some participants. Albeit slight, this limitation should be noted. Small effect sizes are also found throughout the study, which are often frowned upon, however, discovering significant results aligned with theoretical hypotheses in a nonclinical sample suggests that, further along the developmental trajectory for psychotic disorders when differences are more glaring, effect sizes would be more pronounced. Nonetheless, future research could evaluate a similar model in clinical psychosis expressions at a clinical level of psychosis expression.

### 4.5 Conclusions, implications, and future directions

The present findings assessing self- and other-mentalizing separately but simultaneously offered what could be new understanding of the self-other polarities in the psychosis spectrum: self-mentalizing may be the driver behind evidence of impaired mentalization, particularly in those who have experienced intentionally harmful childhood adversity. Mentalization-based treatment has already shown to be effective in reducing psychosis symptoms (111), but these findings further illuminate awareness of which specific mentalization dimensions should be targeted. Indeed, this offers compelling implications for interventions and psychoeducation across the psychosis spectrum. Psychoeducation and interventions focused on self-mentalization should be prioritized particularly in psychosis’ earliest expressions, that is, schizotypy, as interventions that target mentalization in psychosis suggest that early intervention results in better outcomes (111) and contemporary economics demonstrates that it is more beneficial to invest resources early in development in order to capture the full potential of interventions (112, 113).

## Data availability statement

The raw data supporting the conclusions of this article will be made available by the authors, without undue reservation.

## Ethics statement

The studies involving humans were approved by Ethics Committee of the Universitat Autònoma de Barcelona (ref. 5426). The studies were conducted in accordance with the local legislation and institutional requirements. The participants provided their written informed consent to participate in this study.

## Author contributions

JN: Conceptualization, Data curation, Formal analysis, Writing – original draft. PT: Data curation, Formal analysis, Writing – review & editing. TK: Writing – review & editing, Methodology. SB: Writing – review & editing, Conceptualization, Supervision. NB-V: Conceptualization, Supervision, Writing – review & editing, Data curation, Funding acquisition, Project administration, Resources.
